# Association of Low Free T3 with Disease Presence and Activity in Ankylosing Spondylitis

**DOI:** 10.3390/ijms26167862

**Published:** 2025-08-14

**Authors:** Enver Ciftel, Aleksandra Klisic, Bayram Kizilkaya, Osman Cure, Filiz Mercantepe, Sibel Mataraci Karakas, Ana Ninić

**Affiliations:** 1 Department of Endocrinology and Metabolism, Sivas Numune Hospital, 58060 Sivas, Türkiye; enver.ciftel@saglik.gov.tr; 2 Faculty of Medicine, University of Montenegro, 81000 Podgorica, Montenegro; aleksandranklisic@gmail.com; 3 Center for Laboratory Diagnostics, Primary Health Care Center, 81000 Podgorica, Montenegro; 4 Department of Internal Medicine, Recep Tayyip Erdogan University Training and Research Hospital, 53100 Rize, Türkiye; bayram.kizilkaya@saglik.gov.tr; 5 Department of Rheumatology, Faculty of Medicine, Recep Tayyip Erdogan University, 53100 Rize, Türkiye; 6 Department of Endocrinology and Metabolism, Faculty of Medicine, Recep Tayyip Erdogan University, 53100 Rize, Türkiye; 7 Department of Biochemistry, Faculty of Medicine, Recep Tayyip Erdogan University, 53100 Rize, Türkiye; sibel.karakas@erdogan.edu.tr; 8 Department of Medical Biochemistry, Faculty of Pharmacy, University of Belgrade, 11000 Belgrade, Serbia; aninic@pharmacy.bg.ac.rs

**Keywords:** ankylosing spondylitis, FT3, thyroid hormones, disease activity, BASDAI, metabolic markers, inflammation

## Abstract

Ankylosing spondylitis (AS) is a chronic inflammatory disease characterized by axial skeletal involvement and systemic metabolic changes. While inflammation is central to its pathophysiology, the potential role of thyroid hormones, particularly free triiodothyronine (FT3), in disease risk and activity remains underexplored. The objective of this study is to evaluate the relationship between serum FT3 levels and both the presence and clinical activity of AS, while also examining other endocrine-metabolic parameters. In this cross-sectional study, 120 AS patients and 117 healthy controls were assessed. Demographic, anthropometric, hematologic, and biochemical parameters were recorded. Disease activity was determined using the Bath Ankylosing Spondylitis Disease Activity Index (BASDAI), with BASDAI ≥ 4 indicating active disease. Logistic regression models adjusting for age, sex, BMI, and other relevant covariates were applied to identify independent predictors. FT3 levels were significantly lower in AS patients compared to controls (3.25 [3.01–3.58] vs. 3.44 [3.16–3.69] pg/mL, *p* = 0.037) and in patients with BASDAI ≥ 4 versus BASDAI < 4 (3.20 [2.94–3.48] vs. 3.44 [3.19–3.83] pg/mL, *p* = 0.004). The reduction was more evident in women, where it reflected disease presence, whereas in men it was associated with high disease activity. Low FT3 independently predicted both AS (OR 0.50, 95% CI 0.28–0.92, *p* = 0.026) and active disease (OR 0.48, 95% CI 0.24–0.99, *p* = 0.047). Lower HDL-C, BMI, and creatinine, and higher leukocyte counts were also associated with AS, but not with disease activity. Low-normal FT3 is independently associated with both the presence and activity of AS, reflecting disease presence in women and disease activity in men. This is the first study to demonstrate this sex-specific association after adjusting for metabolic parameters and multiple covariates, highlighting FT3’s potential as a marker of inflammation-driven metabolic dysregulation.

## 1. Introduction

Ankylosing spondylitis (AS) is a chronic inflammatory rheumatic disease primarily characterized by axial skeletal involvement and enthesitis. However, growing evidence suggests that its clinical impact extends beyond the musculoskeletal system, involving systemic metabolic and endocrine alterations as well [1,2,3,4,5,6]. In recent years, particular attention has been given to the role of hormonal and metabolic parameters in the pathophysiology of AS [2,7,8,9].

In addition to joint and sacroiliac inflammation, AS has been associated with dysfunction of the hypothalamic–pituitary–adrenal (HPA) axis, subclinical glucocorticoid insufficiency, alterations in thyroid function, and a predisposition to autoimmune thyroid diseases [10,11,12,13,14]. Moreover, abnormalities in peripheral thyroid hormone metabolism consistent with the “non-thyroidal illness syndrome” (NTIS), also known as low T3 syndrome, have been reported in AS patients. NTIS is characterized by reduced serum triiodothyronine (T3) concentrations with normal or low thyroxine (T4) and thyroid-stimulating hormone (TSH) levels, occurring in the absence of intrinsic thyroid disease. This adaptive response is frequently observed in chronic inflammatory and systemic illnesses, where pro-inflammatory cytokines such as IL-1β, IL-6, and TNF-α impair deiodinase activity and hypothalamic–pituitary–thyroid axis function, leading to decreased peripheral conversion of T4 to T3 [15,16,17,18,19,20]. These hormonal alterations are believed to reflect disease severity and poor prognosis in conditions such as malignancy, chronic kidney disease, and heart failure [16,20,21,22,23,24,25,26]. 

In While NTIS has been extensively studied in other chronic inflammatory diseases, its prevalence, clinical significance, and sex-specific patterns in AS remain poorly understood. For instance, Lange et al. reported significantly reduced FT3 and total T3 levels, alongside elevated reverse T3, in female AS patients [15]. Addressing this knowledge gap, the present study investigates the association between low-normal free T3 (FT3) levels and both the presence and clinical activity of AS, integrating metabolic parameters and adjusting for multiple covariates in a large, controlled cohort. We hypothesized that FT3 levels would be inversely associated with disease activity, reflecting inflammation-driven metabolic dysregulation, and could serve as an independent biomarker of AS presence and activity as measured by the Bath Ankylosing Spondylitis Disease Activity Index (BASDAI). By positioning FT3 within the inflammation–hormone axis of AS, our findings may support the incorporation of thyroid hormone assessment into the routine evaluation of AS and underscore the value of a systemic, syndromic approach to disease management.

## 2. Results and Discussion

This study comprehensively evaluated the relationships between FT3, disease presence, and disease activity in AS, integrating baseline clinical and biochemical data, sex- and BASDAI-stratified analyses, correlations, regression models, disease duration, treatment type, and diagnostic performance. The findings provide new insights into the endocrine–immune interplay in AS.

### 2.1. Baseline Demographic, Clinical, and Laboratory Characteristics

The baseline demographic, clinical, and laboratory characteristics of the study groups are presented in Table 1 and Table 2. There were no significant differences in age between patients with AS and healthy controls (*p* > 0.05), ensuring comparability between groups. However, the AS group included a higher proportion of smokers, while exhibiting a lower body mass index (BMI) compared to controls. The finding that the AS group included a higher proportion of smokers and had a lower BMI compared to controls can be explained by several factors. Smoking has been associated with both an increased risk of developing AS and with greater disease severity [27,28,29]. It is thought to enhance systemic inflammation, promote structural damage in the spine, and worsen clinical outcomes. Therefore, a higher prevalence of smoking among AS patients aligns with previous reports. Patients with AS may have a lower BMI due to several mechanisms, including chronic inflammation, increased energy expenditure, and reduced muscle mass associated with long-standing disease [30]. Additionally, pain and stiffness may lead to altered physical activity patterns and changes in body composition. Some patients may adopt lifestyle modifications, such as dietary changes and increased physical activity under medical supervision, which could also contribute to a lower BMI. Overall, this combination of higher smoking rates and lower BMI in AS patients likely reflects both the disease’s pathophysiological mechanisms and behavioral/lifestyle factors associated with chronic illness.

In our study, AS was observed more frequently in women, which contrasts with the traditional view that AS predominantly affects men. Historically, the male-to-female ratio for radiographic AS has been reported as approximately 2–3:1 [31]. However, recent epidemiological data suggest that this gap has narrowed to around 1–1.5:1, largely due to improved imaging techniques and broader diagnostic criteria that now include non-radiographic axial spondyloarthritis, which appears to be more common in women [32].

An important consideration is that the lower reported prevalence of AS in women in earlier studies may have been partly due to underdiagnosis or misclassification. Female patients often present with symptoms that overlap with conditions such as fibromyalgia, leading to delays in diagnosis or alternative diagnoses being made [32]. Therefore, the higher proportion of women with AS in our cohort may reflect not only better recognition of the disease in females but also the possibility that AS is not as strongly male-dominated as previously believed.

Taken together, these findings suggest that sex differences in AS prevalence may be less pronounced than traditionally thought. Our results underscore the need for heightened clinical awareness of AS in women to minimize diagnostic delays and ensure appropriate management.

As expected, inflammatory markers, including C-reactive protein (CRP) and erythrocyte sedimentation rate (ESR), were significantly elevated in AS patients. Hematological parameters revealed higher leukocyte, neutrophil, lymphocyte, monocyte, and platelet counts in the AS group than in controls. In addition, serum vitamin B12 and vitamin D concentrations were higher in AS patients. A plausible explanation for this finding is that AS patients are under regular medical supervision, which likely facilitates the early detection of deficiencies and leads to more frequent supplementation. This increased awareness and proactive management may account for the higher vitamin levels observed in the patient group, whereas creatinine, high-density lipoprotein cholesterol (HDL-C), thyroid-stimulating hormone (TSH), and FT3 levels were lower. These baseline findings establish the context for the subsequent stratified analyses.

### 2.2. Associations Between Clinical, Hematologic, and Biochemical Parameters and AS Presence

To further explore the determinants of AS, we examined the associations between anthropometric, hematologic, and biochemical markers and the presence of AS using univariate and multivariable logistic regression analyses (Table 3).

Univariate regression revealed that several hematologic parameters were positively associated with AS. Higher leukocyte counts were associated with a 38.8% increase in the odds of AS presence (OR 1.388, 95% CI 1.187–1.622, *p* < 0.001). Elevated neutrophil counts were linked to a 32.1% higher likelihood of AS (OR 1.321, 95% CI 1.099–1.589, *p* = 0.003), while increased lymphocyte counts were associated with a more than twofold higher risk (OR 2.034, 95% CI 1.367–3.027, *p* < 0.001). Similarly, high monocyte and platelet counts were significantly related to increased odds of AS (OR 1.535, 95% CI 1.261–1.870, *p* < 0.001; OR 1.084, 95% CI 1.039–1.132, *p* < 0.001, respectively). Elevated ESR was also positively associated with AS (OR 1.078, 95% CI 1.041–1.117, *p* < 0.001). The variance in AS presence explained by these parameters (Nagelkerke R^2^) was 10.2% for leukocytes, 5.1% for neutrophils, 7.3% for lymphocytes, 11.4% for monocytes, 8.5% for platelets, and 10.9% for ESR.

Conversely, negative associations with AS were observed for BMI, creatinine, HDL-C, and FT3 levels. Lower BMI was associated with a 6.5% increase in AS likelihood (OR 0.934, 95% CI 0.887–0.984, *p* = 0.010). Reduced creatinine levels were strongly linked to a higher risk of AS (OR 0.073, 95% CI 0.014–0.383, *p* = 0.002). Similarly, lower HDL-C and FT3 levels were associated with increased odds of AS (OR 0.980, 95% CI 0.962–0.999, *p* = 0.044; OR 0.555, 95% CI 0.332–0.926, *p* = 0.024, respectively). The proportion of variance explained by these negative predictors was 3.9% for BMI, 5.7% for creatinine, 2.5% for HDL-C, and 3.0% for FT3.

All significant predictors identified in the univariate analysis were subsequently included in a multivariable regression model, together with gender and smoking status. In this model, ESR remained a positive independent predictor, whereas BMI, creatinine, and FT3 were independent negative predictors of AS. In contrast, leukocyte count, platelet count, and HDL-C lost statistical significance. The final model accounted for 40.3% of the variance in AS presence, highlighting the combined predictive value of inflammatory markers and metabolic parameters in differentiating AS patients from controls.

Although AS is primarily defined by axial inflammation and enthesitis, systemic metabolic and endocrine alterations are increasingly recognized as part of its broader pathophysiological profile [33,34,35]. Our results support this expanded view, highlighting the potential role of FT3—a thyroid hormone with known metabolic and immunomodulatory effects—as a novel biomarker of immune dysregulation in AS.

Several studies have demonstrated abnormalities in the hypothalamic–pituitary–thyroid (HPT) axis in chronic inflammatory conditions [10,36]. NTIS, or “low T3 syndrome,” is characterized by impaired peripheral conversion of T4 to T3, typically triggered by systemic inflammation. Pro-inflammatory cytokines such as IL-1β, IL-6, and TNF-α inhibit type I deiodinase, the enzyme responsible for T4-to-T3 conversion, resulting in reduced circulating FT3 levels [20]. These cytokines also disrupt HPT axis signaling, suppressing TSH secretion and altering thyroid hormone homeostasis [21,36]. In AS, where IL-6 and TNF-α are key inflammatory mediators, these mechanisms are likely to contribute to the observed reduction in FT3 levels. Additionally, chronic oxidative stress—frequently present in AS—can damage thyroid hormone transport proteins and receptors, impairing cellular responsiveness to T3. Together, these inflammatory and oxidative processes create a metabolic state resembling NTIS, where low FT3 reflects systemic inflammatory stress and metabolic adaptation rather than primary thyroid disease [21,36]. This mechanistic framework supports the role of FT3 as a sensitive indicator of inflammation-driven metabolic alterations in chronic diseases, including AS.

Our findings are consistent with those of Lange et al., who reported decreased FT3 and TT3 levels and elevated reverse T3 levels in women with AS, suggesting an impaired peripheral conversion of T4 [15]. Notably, in our study, low FT3 was not only associated with the diagnosis of AS but also with higher disease activity (BASDAI ≥ 4), even after adjusting for confounders such as gender and smoking status. This suggests that FT3 may serve as a dynamic marker reflecting both immune activation and disease severity.

In our study, FT3 values in AS patients were mostly within the laboratory reference range but tended to cluster toward its lower end. We considered these values as ‘low normal,’ reflecting inflammation-driven alterations in thyroid hormone metabolism without overt hypothyroidism. In contrast, ‘abnormal’ FT3 would refer to values below the lower reference limit, which were not observed in our cohort. This distinction is clinically relevant, as even low-normal FT3 may signal metabolic adaptation to chronic inflammation rather than primary thyroid disease [21,36].

In addition to FT3, we identified other metabolic and hematologic parameters associated with AS. Lower HDL-C, BMI, and serum creatinine levels were independently associated with AS, while leukocyte count was significantly higher in AS patients. These findings are in line with previous studies reporting altered lipid metabolism, reduced muscle mass, and systemic inflammation in AS [30,37,38,39,40,41]. However, unlike FT3, none of these variables were associated with disease activity, further emphasizing the distinctive role of thyroid hormone metabolism in the pathogenesis of AS.

### 2.3. Comparison of AS Patients by Disease Activity

The demographic, clinical, anthropometric, and biochemical characteristics of patients with AS were compared based on a BASDAI threshold of four, dividing the patients into low disease activity (BASDAI < 4) and high disease activity (BASDAI ≥ 4) groups (Table 4 and Table 5).

There were no significant differences between the two groups in terms of age, BMI, waist circumference, or comorbidities, indicating overall comparability. However, gender distribution differed between groups, with a higher proportion of women in the BASDAI ≥ 4 group, suggesting that female patients may report higher disease activity or experience a distinct symptom burden. The studies report that women experience higher pain intensity and symptom burden than men, even when disease severity is comparable. In axial spondyloarthritis—including ankylosing spondylitis—female patients consistently report higher disease activity and pain scores than male patients [42]. Broad reviews of chronic pain conditions similarly confirm that women display greater pain sensitivity, lower pain thresholds, and more frequent or severe pain experiences compared to men [43]. These sex-based differences likely reflect a combination of biological pathways (e.g., hormone-mediated differences in nociception), neuro-immune mechanisms, and psychosocial factors that amplify pain perception in women.

The use of non-steroidal anti-inflammatory drugs (NSAIDs) was more frequent among patients with high disease activity (*p* = 0.004). The higher frequency of NSAID use among patients with high disease activity likely reflects the fact that NSAIDs are first-line agents for symptom control in AS, and their use typically increases during periods of heightened inflammation and pain. This finding is consistent with treatment patterns reported in previous studies, where patients with active disease required more intensive NSAID therapy to achieve symptomatic relief [44,45]. Therefore, increased NSAID use in this subgroup may not be a cause of higher disease activity but rather a consequence of it.

Hematological and biochemical analysis revealed that only total protein and FT3 levels were significantly lower in patients with a BASDAI ≥ 4 compared to those with a BASDAI < 4 (*p* = 0.039, *p* = 0.004, respectively). This supports the association between low FT3 and increased disease activity. In contrast, inflammatory markers such as CRP and ESR, although numerically higher in the BASDAI ≥ 4 group, did not differ significantly, likely reflecting treatment effects or variability in systemic inflammatory markers.

**Figure 1:**
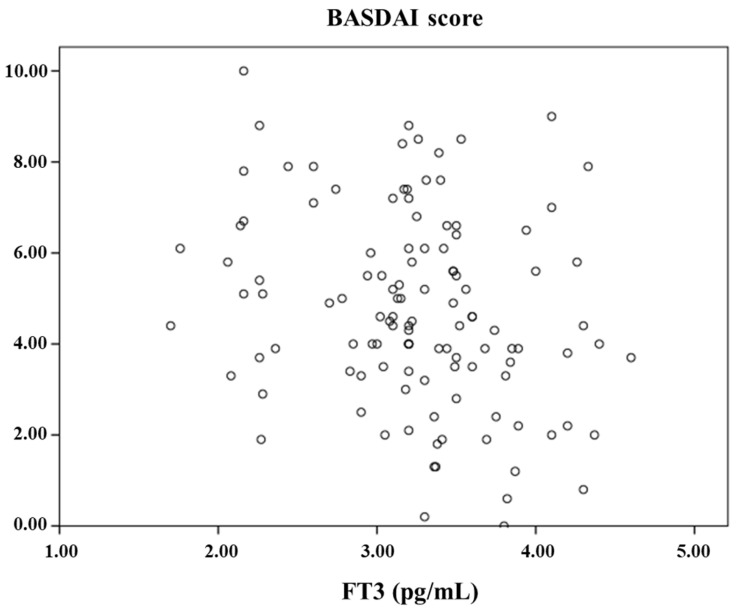
Scatter plot representing the relationship between FT3 and BASDAI score.

Additionally, the relationships between FT3, BASDAI score, and inflammatory markers were examined using Spearman correlation analysis. FT3 showed a significant negative correlation with BASDAI score, CRP, and ESR, and a positive correlation with lymphocyte count. These findings indicate that lower FT3 levels are associated with higher disease activity. CRP exhibited positive correlations with ESR, WBC, and lymphocyte count. As expected, WBC and lymphocyte counts were also positively correlated. The correlation coefficients for these variables are presented in the correlation matrix shown in Table 6. The scatter plot (Figure 1) visually supports these findings, showing an inverse modest relationship between FT3 concentrations and BASDAI scores.

We investigated the associations between anthropometric variables, hematologic and biochemical markers, and BASDAI score ≥ 4 in AS patients and controls (Table 7). Univariate binary regression analysis revealed only negative associations of uric acid and FT3 with BASDAI score ≥ 4. Low levels of uric acid were related to 26.6% increased odds for BASDAI score ≥ 4 (OR 0.734, 95%CI 0.542–0.994, *p* = 0.046). Low levels of FT3 were related to 58.6% increased odds for BASDAI score ≥ 4 (OR 0.414, 95%CI 0.206–0.831, *p* = 0.013). The Nagelkerke R^2^ for uric acid and FT3 was able to explain the variation in the BASDAI score ≥ 4 by 4.7% and 3.0%, respectively. Both significant predictors of univariable analysis were tested together with gender in a multivariable analysis (Table 7). Only FT3 kept its independent negative association with the BASDAI score ≥ 4. The model was able to explain the 18.9% variation in BASDAI score ≥ 4.

### 2.4. Sex- and BASDAI-Stratified Analyses of Clinical and Biochemical Characteristics (Supplementary Tables S1–S8)

Sex-stratified analyses revealed distinct patterns. In women, FT3 levels were significantly lower than in female controls, but did not vary according to BASDAI. In men, FT3 levels were not significantly different from male controls, but decreased markedly with higher BASDAI scores. Men with BASDAI ≥ 4 also had lower HDL-C and vitamin B12 levels, patterns absent in women. These results suggest that in women, FT3 reduction reflects disease presence, while in men, it serves as an indicator of disease activity.

This sex-dependent response may reflect differences in immune–endocrine regulation, where inflammatory signals have distinct effects on thyroid hormone metabolism across genders [46,47]. Additionally, BASDAI scores in women may be influenced by factors beyond measurable inflammation, including pain perception and hormonal modulation [42,43].

In men, FT3 and other biochemical parameters may serve as adjunct markers for assessing disease activity, while in women BASDAI scores should be interpreted with caution as they may not directly correspond to inflammatory biochemical profiles. These observations are consistent with previous studies emphasizing the importance of a sex-sensitive approach to evaluating disease activity in AS [42].

### 2.5. Effects of Disease Duration and Treatment Type on Thyroid Function

To further evaluate factors that might influence thyroid hormone status in AS, patients were stratified by disease duration and the type of treatment. The analyses are presented in Supplementary Tables S9 and S10.

Because the therapeutic response in AS is typically assessed at the 6-month mark, patients were classified into two groups: those with a disease duration ≤6 months and those with a disease duration >6 months. Comparisons revealed no significant differences in TSH, FT4, or FT3 levels between these groups (Supplementary Table S9). FT3 levels were nearly identical in both groups, indicating that the chronicity of AS does not influence thyroid hormone parameters. This finding supports the notion that alterations in FT3 observed in AS patients are primarily associated with inflammatory activity, rather than the duration of disease [21]. Similar observations have been reported in other chronic inflammatory disorders, where thyroid hormone changes are driven by active inflammation rather than disease chronicity [16,22,23,48].

Patients were also compared according to the treatment received. All AS patients were treated either with NSAIDs or anti-TNF-α biologic agents; none were on DMARDs or corticosteroids. The comparison between the NSAID and anti-TNF-α groups demonstrated no significant differences in TSH, FT4, or FT3 levels (Supplementary Table S10). These findings suggest that the type of treatment does not significantly impact thyroid hormone levels, and that the observed FT3 alterations are more likely to reflect the underlying inflammatory state rather than treatment effects. Previous studies have similarly shown that anti-TNF therapy effectively reduces inflammation but does not directly alter thyroid hormone metabolism [49].

These supplementary analyses indicate that FT3 alterations in AS are independent of both disease duration and treatment type. The reduction in FT3 observed in AS patients is likely attributable to inflammation-driven metabolic adaptations, consistent with the mechanisms underlying non-thyroidal illness syndrome [21]. The lack of effect from treatment type further supports that FT3 is a marker of disease-related processes rather than a treatment-modifiable parameter.

### 2.6. Diagnostic Performance of FT3

Receiver operating characteristic curve (ROC) analyses demonstrated that FT3 has poor discriminatory ability for distinguishing AS patients from controls (AUC—area under the curve = 0.579, *p* = 0.037) and low accuracy for identifying patients with high vs. low disease activity (AUC = 0.659, *p* = 0.004), (Table S11). No clinically meaningful cut-off was identified, making a “number needed to test” calculation infeasible. Thus, FT3 is not a diagnostic biomarker, although it may have value as an adjunct marker of inflammatory metabolic changes.

### 2.7. Clinical Significance and Mechanistic Considerations of FT3 in Ankylosing Spondylitis

This study demonstrates that FT3 levels, while remaining within the normal reference range, are consistently lower in patients with AS compared to healthy controls. Although the absolute differences were modest, this reduction likely reflects a metabolic adaptation to chronic inflammation rather than primary thyroid dysfunction. Small decreases in FT3, even within the normal range, may signal systemic inflammatory burden and altered metabolic regulation, as observed in other chronic inflammatory diseases [21].

The pattern of FT3 alteration was sex-dependent. In women, FT3 was lower than in controls regardless of disease activity, suggesting that its reduction is an early and persistent feature of the disease. In men, FT3 levels were similar to controls but decreased significantly in patients with high disease activity, indicating that FT3 is more responsive to inflammation during active disease phases. These findings align with evidence that immune–endocrine interactions differ between sexes in chronic inflammation and may contribute to variations in disease expression [50].

Additional analyses confirmed that FT3 changes were not influenced by disease duration or treatment type, supporting the notion that these alterations are driven by inflammatory mechanisms rather than therapy or chronicity. Regression analyses further demonstrated that low FT3 independently predicted high disease activity, although its overall predictive power remained modest. ROC analyses showed poor diagnostic performance, confirming that FT3 is not a suitable diagnostic biomarker.

From a mechanistic standpoint, thyroid hormones modulate both innate and adaptive immune responses. FT3 influences T cell differentiation, macrophage activation, and cytokine production, and its deficiency may promote a pro-inflammatory phenotype [50,51]. The presence of thyroid hormone receptors and transporters on immune cells provides a molecular basis for this interaction [50]. The reduction in FT3 observed in AS is consistent with the concept of non-thyroidal illness syndrome, where pro-inflammatory cytokines (IL-1β, IL-6, and TNF-α) inhibit the peripheral conversion of T4 to T3, suppress hypothalamic–pituitary–thyroid axis activity, and alter thyroid hormone receptor sensitivity. These pathways explain why low FT3 may reflect inflammatory-driven metabolic dysregulation in AS [21,36,52].

### 2.8. Clinical Implications

Although FT3 lacks diagnostic utility due to its limited accuracy, it may serve as a supplementary marker of inflammatory metabolic status, particularly in patients where conventional inflammatory markers such as CRP and ESR provide incomplete information. In men with active disease, FT3 reduction independently predicts high disease activity, whereas in women, it reflects the presence of disease without correlating with activity levels—possibly due to additional non-inflammatory influences on disease assessment.

FT3 testing is not currently recommended in routine AS management. However, in selected scenarios—such as patients with high disease activity, persistent systemic symptoms, or discordant clinical and laboratory findings—its measurement may offer additional clinical insight. Based on these findings, an exploratory algorithm (Figure S1) could be considered, proposing FT3 testing as an adjunct tool; however, this approach remains hypothetical and requires validation in future prospective studies.

### 2.9. Study Limitations, Therapeutic Perspective, and Future Directions

This study has several limitations that should be considered when interpreting the findings. First, its cross-sectional design precludes establishing causality, making it unclear whether low FT3 levels actively contribute to AS pathogenesis or merely reflect underlying inflammation. Although our results suggest that low FT3 is associated with higher disease activity and may serve as a marker of systemic inflammation, the design of the study does not allow conclusions about its role in guiding treatment decisions. At present, FT3 should not be regarded as an independent tool for therapy adjustment. Longitudinal and interventional studies are required to determine whether monitoring FT3 levels could contribute to treatment optimization in AS.

Second, thyroid autoantibodies were not measured, limiting the ability to differentiate non-thyroidal illness from subclinical autoimmune thyroid disease. Third, the lack of experimental assays, such as deiodinase activity and immune cell profiling, restricted a deeper understanding of the biological consequences of altered FT3 levels. In addition, menstrual status and hormonal therapy use in female participants were not recorded, introducing possible residual confounding. Although patients receiving medications other than NSAIDs or TNF-α inhibitors were excluded, the influence of other unmeasured factors on thyroid hormone levels cannot be completely ruled out.

Furthermore, the study was conducted at a single center, which may limit the generalizability of the findings to other AS populations. Lastly, while we identified significant associations between low FT3 and both the presence and activity of AS, the study does not provide direct mechanistic evidence. Low FT3 in AS appears to reflect inflammation-driven metabolic alterations rather than primary thyroid dysfunction. Current evidence does not support FT3 as a direct treatment target. Instead, the effective control of systemic inflammation through standard therapies, including TNF-α inhibitors, may indirectly normalize thyroid hormone metabolism. Future longitudinal, mechanistic, and interventional studies are needed to clarify whether monitoring FT3 can contribute to treatment decision-making and whether interventions that modulate thyroid hormone metabolism might influence disease outcomes.

## 3. Materials and Methods

### 3.1. Study Design and Participants

This cross-sectional, retrospective study was conducted to investigate the associations between endocrine–metabolic–inflammation parameters and AS. A total of 237 participants were included: 120 patients diagnosed with AS according to the Assessment in Spondylarthritis International Society (ASAS) criteria [53], and 117 age-matched healthy controls without known rheumatologic or endocrine diseases. All participants were recruited from the outpatient clinics of a tertiary care university hospital between January 2023 and December 2024.

Exclusion criteria included the following:

- Known thyroid disease or thyroid hormone replacement therapy.
- Individuals taking medications known to influence thyroid function, including amiodarone and propranolol.
- Chronic kidney or liver disease.
- Malignancy.
- Uncontrolled diabetes mellitus and hypertension.
- Acute infection or chronic inflammatory diseases other than AS.
- Patients receiving medications other than non-steroidal anti-inflammatory drugs (NSAIDs) or TNF-α inhibitors were excluded from the study. All AS patients were receiving either NSAIDs and/or TNF-α inhibitors as part of their treatment regimen. The TNF-α inhibitors used included infliximab, etanercept, adalimumab, certolizumab, and golimumab. None of the patients were treated with corticosteroids, conventional immunosuppressive agents, or DMARDs.

Ethical approval was obtained from the Institutional Review Board of Recep Tayyip Erdogan University local ethics commission (Approval Number: 2024/310, Approval date: 26 December 2024). As this is a retrospective study, an informed consent form is unnecessary, and the study was conducted in accordance with the Declaration of Helsinki.

### 3.2. Clinical and Anthropometric Assessments

Detailed demographic data (age, sex, smoking and alcohol status, and comorbidities) and anthropometric measurements (height, weight, BMI, and waist circumference) were recorded. BMI was calculated as weight in kilograms divided by the square of height in meters (kg/m^2^). Disease duration, Bath Ankylosing Spondylitis Disease Activity Index (BASDAI), and Bath Ankylosing Spondylitis Functional Index (BASFI) were documented in all AS patients [54]. BASDAI ≥ 4 was used as the cutoff to define active disease.

### 3.3. Laboratory Measurements

All blood samples were collected between 08:00 and 10:00 a.m. after an overnight fast of at least 8 h, with participants resting in a seated position for 10 min prior to venipuncture to minimize variability in hormone levels. The following parameters were measured using standardized automated techniques at the institutional laboratory:

- Hematologic markers: white blood cell count, neutrophils, lymphocytes, monocytes, and platelet count.
- Biochemical markers: glucose, hemoglobin A1c (HbA1c), urea, creatinine, uric acid, total protein, C-reactive protein (CRP), lipid profile (total cholesterol, high-density lipoprotein cholesterol (HDL-C), low-density lipoprotein cholesterol (LDL-C), and triglycerides), vitamin B12, folate, vitamin D, and magnesium.
- Thyroid function tests: TSH, free T4 (FT4), and free T3 (FT3). Serum FT3, FT4, and TSH concentrations were measured using an electrochemiluminescence immunoassay (ECLIA) on a Cobas e601 analyzer (Roche Diagnostics, Mannheim, Germany). The intra-assay and inter-assay coefficients of variation (CVs) were <5% and <8%, respectively. The reference ranges for FT3, FT4, and TSH were 2.0–4.4 pg/mL, 0.93–1.7 ng/dL, and 0.34–4.2 µIU/mL, respectively.

All assays were performed using commercially available kits according to the manufacturers’ protocols.

### 3.4. Statistical Analysis

The Kolmogorov–Smirnov test was used to evaluate the distribution of the data. Variables with a normal distribution were expressed as mean ± standard deviation (SD) and analyzed using the Student’s *t*-test. Non-normally distributed data were summarized using the median and interquartile range (IQR), with group differences assessed using the Mann–Whitney *U* test. For categorical variables, results were presented as counts, with group comparisons performed using the Chi-square test for contingency tables. Spearman correlation analysis was used to test relationships between FT3, BASDAI score, and inflammation markers. Data were given as correlation coefficients (ρ).

Binary logistic regression models were used in both univariable and multivariable settings to examine the association between anthropometric, hematologic, and biochemical parameters (independent continuous variables) and the presence of ankylosing spondylitis (binary outcome: 0 for control, 1 for ankylosing spondylitis) and the BASDAI score cut-off (binary outcome: 0 for BASDAI < 4 and 1 for BASDAI ≥ 4). In the multivariable analyses, only the continuous variables significant in univariable logistic regression analysis and categorical variables that showed significant differences between tested groups were considered as potential confounders. The data from the binary regression analysis were expressed as an odds ratio (OR) and 95% confidence interval. The extent to which these factors were responsible for the ankylosing spondylitis risk and risk for BASDAI ≥ 4 was quantified using the Nagelkerke R^2^. Receiver operating characteristic (ROC) curve analysis was used with the purpose of testing the discriminatory potential of FT3 regarding ankylosing spondylitis risk and risk for BASDAI ≥ 4. Data were expressed as the area under the curve (AUC) and 95% confidence interval.

All statistical analyses were performed using SPSS software (version 22.0; SPSS Inc., Chicago, IL, USA), with the significance level set at *p* < 0.05. For type II error, β was set to ≤0.2. To control the false discovery rate, we used the Benjamini–Hochberg procedure. The Benjamini–Hochberg critical value was calculated for each individual *p*-value. We found the largest *p*-value that was less than the critical value. Every *p*-value that was smaller than this *p*-value was considered to be significant.

## 4. Conclusions

FT3 levels are reduced in AS despite remaining within the normal range, showing a sex-dependent pattern. In women, lower FT3 primarily reflects the presence of the disease, whereas in men it is more closely associated with disease activity. These alterations are not influenced by disease duration or treatment type, suggesting they are driven by underlying inflammatory processes. Although FT3 has limited diagnostic value, its measurement may provide additional insight into the inflammatory metabolic status of patients, particularly in men with active disease. This is the first study in AS to identify a sex-specific association between low-normal FT3 and both disease presence and activity after adjusting for metabolic parameters and multiple covariates, highlighting its potential as a marker of inflammation-driven metabolic dysregulation. Future prospective studies should explore its prognostic significance and potential role in complementing existing composite indices for AS assessment.

## Figures and Tables

**Table 1:** General data of the examined population.

	Control Group n = 117	Ankylosing Spondylitis n = 120	*p*
N (male/female)	56/61	74/46	0.033
Age, years	39.00 (30.00–47.00)	37.00 (31.00–47.00)	0.949
BMI, kg/m^2 #^	28.38 ± 5.12	26.59 ± 5.21	0.008
Waist circumference, cm	95.00 (84.00–102.50)	92.00 (86.50–104.00)	0.943
Diabetes mellitus (no/yes)	117/0	119/1	0.322
Coronary artery disease (no/yes)	117/0	119/1	0.322
Hypertension (no/yes)	114/3	116/4	0.727
Hyperlipidemia (no/yes)	117/0	119/1	0.322
Alcohol drinking status (no/yes)	113/4	113/7	0.377
Smoking status (no/yes)	86/31	71/49	0.020
Disease duration, months	/	6.00 (4.00–10.00)	/
NSAID/Anti-TNF-α agents	/	45/75	/
BASDAI score	/	4.75 ± 2.15	/
BASFI score	/	3.10 (1.20–5.00)	/

Data are presented as median (IQR) and compared with the Mann–Whitney *U* test. ^#^ Normally distributed data are presented as mean ± standard deviation and compared with the Student’s *t*-test. Categorical variables are presented as absolute frequencies and compared by Chi-square test for contingency tables. Abbreviations: BMI, body mass index; NSAID, non-steroidal anti-inflammatory drugs; TNF-α, tumor necrosis factor-alpha; BASDAI, Bath Ankylosing Spondylitis Disease Activity Index; and BASFI, Bath Ankylosing Spondylitis Functional Index.

**Table 2:** Hematologic and biochemical markers of examined population.

	Control Group	Ankylosing Spondylitis	*p*
WBC × 1000	6.70 (5.45–7.73)	7.59 (6.58–9.06)	<0.001
Neutrophil count × 1000	3.62 (3.07–4.97)	4.67 (3.44–5.53)	0.002
Lymphocyte count × 1000	2.07 (1.74–2.48)	2.39 (1.96–2.96)	<0.001
Monocyte count × 100	3.80 (3.20–4.80)	4.75 (3.85–5.80)	<0.001
Platelet count × 10,000	26.00 (21.00–30.00)	28.00 (25.00–33.50)	<0.001
Glucose (mg/dL)	92.00 (88.00–97.00)	91.00 (85.00–98.00)	0.237
HbA1c, %	5.60 (5.30–5.90)	5.60 (5.20–5.85)	0.293
Urea (mg/dL)	26.00 (21.00–31.00)	27.00 (23.00–32.00)	0.387
Creatinine (mg/dL)	0.79 (0.70–0.90)	0.70 (0.60–0.90)	0.005
Uric acid (mg/dL)	4.70 (3.90–6.00)	5.00 (4.20–5.95)	0.408
Total protein (g/dL)	7.56 (7.37–7.80)	7.70 (7.05–8.00)	0.266
CRP (mg/L)	1.83 (0.84–3.53)	5.00 (2.00–8.50)	<0.001
ESR (mm/hour)	7.00 (4.00–12.00)	12.00 (4.00–20.00)	0.001
Total cholesterol ^#^ (mg/dL)	213.17 ± 43.44	205.50 ± 37.73	0.134
HDL-C (mg/dL)	52.00 (44.00–61.50)	49.00 (41.00–57.00)	0.030
LDL-C ^#^ (mg/dL)	133.96 ± 38.08	129.45 ± 32.51	0.332
Triglycerides (mg/dL)	106.00 (75.00–173.00)	118.50 (81.00–167.00)	0.401
Vitamin B12 (pg/mL)	304.00 (258.00–353.00)	331.00 (265.00–413.50)	0.018
Folate (ng/mL)	9.00 (7.32–11.74)	8.00 (6.30–11.00)	0.111
Vitamin D (ng/mL)	8.60 (6.00–13.73)	10.00 (7.00–16.00)	0.045
Magnesium (mg/dL)	2.00 (1.92–2.10)	1.98 (1.90–2.09)	0.060
TSH (mIU/L)	2.02 (1.47–3.00)	1.62 (1.21–2.76)	0.021
FT4 (ng/dL)	1.16 (1.05–1.26)	1.15 (1.04–1.31)	0.656
FT3 (pg/mL)	3.44 (3.16–3.69)	3.25 (3.01–3.58)	0.037

Data are presented as median (interquartile range) and compared with the Mann–Whitney *U* test. ^#^ Normally distributed data are presented as mean ± standard deviation and compared with the Student’s *t*-test. Abbreviations: WBC, white blood cell count; HbA1c, hemoglobin A1c; CRP, C-reactive protein; ESR, erythrocyte sedimentation rate; HDL-C, high-density lipoprotein cholesterol; LDL-C, low-density lipoprotein cholesterol; TSH, thyroid-stimulating hormone; FT4, free thyroxine; and FT3, free triiodothyronine.

**Table 3:** Estimated odds ratios after binary regression analysis for associations of demographic and laboratory markers with ankylosing spondylitis.

Single Predictors	OR (95%CI)	*p*	Nagelkerke R^2^
Age, years	0.998 (0.975–1.022)	0.887	0.000
BMI, kg/m^2^	0.934 (0.887–0.984)	0.010	0.039
Waist circumference, cm	1.001 (0.982–1.020)	0.918	0.000
WBC count	1.388 (1.187–1.622)	<0.001	0.102
Neutrophil count	1.321 (1.099–1.589)	0.003	0.051
Lymphocyte count	2.034 (1.367–3.027)	<0.001	0.073
Monocyte count	1.535 (1.261–1.870)	<0.001	0.114
Platelet count	1.084 (1.039–1.132)	<0.001	0.085
Glucose (mg/dL)	0.976 (0.953–1.000)	0.051	0.024
HbA1c, %	0.586 (0.316–1.086)	0.090	0.025
Urea (mg/dL)	1.020 (0.985–1.056)	0.267	0.007
Creatinine (mg/dL)	0.073 (0.014–0.383)	0.002	0.057
Uric acid (mg/dL)	0.026 (0.850–1.225)	0.774	0.000
Total protein (g/dL)	1.124 (0.655–1.928)	0.671	0.009
CRP (mg/L)	1.026 (0.987–1.067)	0.197	0.013
ESR (mm/hour)	1.078 (1.041–1.117)	<0.001	0.109
Total cholesterol (mg/dL)	0.995 (0.989–1.002)	0.135	0.013
HDL-C (mg/dL)	0.980 (0.962–0.999)	0.044	0.025
LDL-C (mg/dL)	0.996 (0.989–1.004)	0.331	0.005
Triglycerides (mg/dL)	1.000 (0.996–1.003)	0.975	0.000
Vitamin B12 (pg/mL)	1.002 (1.000–1.004)	0.097	0.018
Folate (ng/mL)	0.977 (0.917–1.040)	0.468	0.003
Vitamin D (ng/mL)	1.018 (0.984–1.052)	0.304	0.006
Magnesium (mg/dL)	0.308 (0.070–1.358)	0.120	0.005
TSH (mIU/L)	0.852 (0.701–1.036)	0.109	0.015
FT4 (ng/dL)	1.526 (0.436–5.339)	0.508	0.003
FT3 (pg/mL)	0.555 (0.332–0.926)	0.024	0.030
	Adjusted		
Predictors in Models	OR (95% CI)	*p*	Nagelkerke R^2^
BMI (kg/m^2^)	0.899 (0.840–0.962)	0.002	0.403
WBC count	1.226 (0.989–1.520)	0.063	
Platelet count	1.032 (0.976–1.092)	0.271	
Creatinine (mg/dL)	0.005 (0.000–0.081)	<0.001	
ESR (mm/hour)	1.083 (1.035–1.134)	0.001	
HDL-C (mg/dL)	0.983 (0.958–1.010)	0.211	
FT3 (pg/mL)	0.423 (0.218–0.822)	0.011	

Data are given as OR (95% CI). Models included each marker and categorical variables (gender and smoking status). Abbreviations: BMI, body mass index; WBC, white blood cell count; HbA1c, hemoglobin A1c; CRP, C-reactive protein; ESR, erythrocyte sedimentation rate; HDL-C, high-density lipoprotein cholesterol; LDL-C, low-density lipoprotein cholesterol; TSH, thyroid-stimulating hormone; FT4, free thyroxine; FT3, free triiodothyronine; and OR, odds ratio.

**Table 4:** General data of patients with ankylosing spondylitis according to BASDAI score.

	BASDAI Score < 4 n = 44	BASDAI Score ≥ 4 n = 76	*p*
N (male/female)	33/11	42/34	0.026
Age, years	38.00 (30.02–44.00)	37.00 (31.00–49.00)	0.653
BMI, kg/m^2 #^	25.61 ± 4.50	27.21 ± 5.51	0.105
Waist circumference, cm	92.50 (87.00–101.00)	91.00 (86.00–106.00)	0.978
Diabetes mellitus (no/yes)	44/0	75/1	0.448
Coronary artery disease (no/yes)	44/0	75/1	0.448
Hypertension (no/yes)	42/2	73/2	0.564
Hyperlipidemia (no/yes)	44/0	75/1	0.448
Alcohol drinking status (no/yes)	40/4	72/4	0.407
Smoking status (no/yes)	23/21	48/29	0.279
Disease duration, months	7.00 (5.00–10.50)	5.00 (3.00–10.00)	0.095
NSAID/Anti-TNF-α biological agents	9/35	36/41	0.004
BASDAI score	2.85 (1.90–3.55)	5.60 (4.60–7.20)	<0.001
BASFI score	1.40 (0.55–3.80)	4.00 (2.25–6.05)	<0.001

Data are presented as median (interquartile range) and compared with the Mann–Whitney *U* test. ^#^ Normally distributed data are presented as mean ± standard deviation and compared with the Student’s *t*-test. Categorical variables are presented as absolute frequencies and compared by Chi-square test for contingency tables. Abbreviations: BMI, body mass index; NSAID, non-steroidal anti-inflammatory drugs; TNF-α-tumor necrosis factor-alpha; BASDAI, Bath Ankylosing Spondylitis Disease Activity Index; and BASFI, Bath Ankylosing Spondylitis Functional Index.

**Table 5:** Hematologic and biochemical markers of patients with ankylosing spondylitis according to BASDAI score.

	BASDAI Score < 4 n = 44	BASDAI Score ≥ 4 n = 76	*p*
WBC count × 1000	7.45 (6.53–8.69)	7.68 (6.63–9.21)	0.527
Neutrophil count × 1000	4.29 (3.23–5.41)	4.75 (3.59–5.62)	0.286
Lymphocyte count × 1000	2.51 (1.99–3.01)	2.31 (1.96–2.96)	0.865
Monocyte count × 100	4.80 (4.15–5.95)	4.60 (3.80–5.80)	0.329
Platelet count × 10,000	28.00 (24.00–32.50)	28.00 (26.00–34.0)	0.389
Glucose (mg/dL)	91.00 (84.00–99.00)	91.00 (85.00–97.00)	0.929
HbA1c, %	5.60 (5.20–5.80)	5.50 (5.20–5.90)	0.518
Urea (mg/dL)	27.00 (23.50–29.50)	26.00 (21.00–32.00)	0.493
Creatinine (mg/dL)	0.80 (0.70–0.90)	0.70 (0.60–0.90)	0.069
Uric acid (mg/dL)	5.15 (4.45–6.00)	4.80 (4.00–5.90)	0.065
Total protein (g/dL)	8.00 (7.60–8.00)	7.60 (7.00–8.00)	0.039
CRP (mg/L)	4.50 (1.75–8.50)	5.00 (2.00–9.00)	0.409
ESR (mm/hour)	10.50 (4.00–19.00)	13.00 (4.00–22.00)	0.264
Total cholesterol ^#^ (mg/dL)	207.00 ± 36.71	205.99 ± 42.92	0.896
HDL-C (mg/dL)	50.50 (43.50–59.00)	47.00 (40.00–56.00)	0.135
LDL-C ^#^ (mg/dL)	128.09 ± 32.51	131.77 ± 37.77	0.590
Triglycerides (mg/dL)	117.00 (74.00–188.50)	119.00 (86.00–162.00)	0.765
Vitamin B12 (pg/mL)	347.00 (278.00–416.00)	326.00 (259.00–412.00)	0.135
Folate (ng/mL)	8.00 (6.75–13.00)	8.00 (6.00–11.00)	0.568
Vitamin D (ng/mL)	12.00 (8.50–16.50)	10.00 (6.00–15.00)	0.168
Magnesium (mg/dL)	2.00 (1.87–2.07)	1.96 (1.90–2.10)	0.659
TSH (mIU/L)	1.60 (1.20–2.32)	1.64 (1.21–2.80)	0.708
FT4 (ng/dL)	1.21 (1.07–1.32)	1.12 (1.04–1.29)	0.268
FT3 (pg/mL)	3.44 (3.19–3.83)	3.20 (2.94–2.48)	0.004

Data are presented as median (interquartile range) and compared with the Mann–Whitney *U* test. ^#^ Normally distributed data are presented as mean ± standard deviation and compared with the Student’s *t*-test. Abbreviations: WBC, white blood cell count; HbA1c, hemoglobin A1c; CRP, C-reactive protein; HDL-C, high-density lipoprotein cholesterol; LDL-C, low-density lipoprotein cholesterol; TSH, thyroid-stimulating hormone; FT4, free thyroxine; and FT3, free triiodothyronine.

**Table 6:** Correlation matrix of relationships between FT3, BASDAI, and inflammatory markers.

FT3 (pg/mL)	1.000					
BASDAI score	−0.243 **	1.000				
CRP (mg/L)	−0.152 *	0.152	1.000			
ESR (mm/hour)	−0.259 ***	0.144	0.482 ***	1.000		
WBC count	0.071	0.045	0.421 ***	0.139 *	1.000	
Lymphocyte count	0.145 *	−0.030	0.129 *	−0.017	0.503 ***	1.000
Correlation matrix	FT3 (pg/mL)	BASDAI score	CRP (mg/L)	ESR (mm/hour)	WBC count	Lymphocyte count

Data are presented as Spearman correlation coefficients (ρ). *** *p* < 0.001; ** *p* < 0.01; and * *p* < 0.05. Abbreviations: FT3, free triiodothyronine; BASDAI, BASDAI, Bath Ankylosing Spondylitis Disease Activity Index; CRP, C-reactive protein; ESR, erythrocyte sedimentation rate; and WBC, white blood cell count.

**Table 7:** Estimated odds ratios after binary regression analysis for BASDAI score as a dependent variable.

Single Predictors	OR (95%CI)	*p*	Nagelkerke R^2^
Age, years	1.012 (0.977–1.049)	0.514	0.005
BMI, kg/m^2^	1.065 (0.986–1.149)	0.108	0.031
Waist circumference, cm	1.008 (0.978–1.040)	0.595	0.003
WBC count	1.080 (0.878–1.328)	0.467	0.006
Neutrophil count	1.140 (0.882–1.474)	0.317	0.012
Lymphocyte count	1.017 (0.626–1.654)	0.944	0.000
Monocyte count	0.875 (0.692–1.106)	0.263	0.014
Platelet count	1.025 (0.972–1.082)	0.356	0.010
Glucose (mg/dL)	1.001 (0.961–1.042)	0.981	0.000
HbA1c, %	0.681 (0.258–1.801)	0.439	0.007
Urea (mg/dL)	0.987 (0.942–1.035)	0.601	0.003
Creatinine (mg/dL)	0.117 (0.010–1.295)	0.080	0.035
Uric acid (mg/dL)	0.734 (0.542–0.994)	0.046	0.047
Total protein (g/dL)	1.029 (0.925–1.145)	0.604	0.005
CRP (mg/L)	1.047 (0.975–1.125)	0.208	0.020
ESR (mm/hour)	1.025 (0.985–1.067)	0.216	0.018
Total cholesterol (mg/dL)	0.999 (0.990–1.009)	0.895	0.000
HDL-C (mg/dL)	0.980 (0.952–1.009)	0.170	0.021
LDL-C (mg/dL)	1.003 (0.992–1.014)	0.587	0.005
Triglycerides (mg/dL)	0.999 (0.993–1.005)	0.717	0.001
Vitamin B12 (pg/mL)	0.997 (0.994–1.000)	0.057	0.042
Folate (ng/mL)	0.973 (0.897–1.055)	0.507	0.005
Vitamin D (ng/mL)	0.965 (0.918–1.014)	0.160	0.023
Magnesium (mg/dL)	1.740 (0.235–12.888)	0.588	0.003
TSH (mIU/L)	1.021 (0.773–1.349)	0.882	0.000
FT4 (ng/dL)	0.421 (0.075–2.370)	0.327	0.011
FT3 (pg/mL)	0.414 (0.206–0.831)	0.013	0.030
	Adjusted		
Predictors in Models	OR (95% CI)	*p*	Nagelkerke R^2^
Uric acid (mg/dL)	0.891 (0.605–1.312)	0.559	0.189
FT3 (pg/mL)	0.484 (0.232–0.999)	0.049	

Data are given as OR (95% CI). Models included each marker and categorical variables (gender, NSAID/Anti-TNF-α agents). Abbreviations: BMI, body mass index; WBC, White blood cell count; HbA1c, hemoglobin A1c; CRP, C-reactive protein; ESR, erythrocyte sedimentation rate; HDL-C, high-density lipoprotein cholesterol; LDL-C, low-density lipoprotein cholesterol; TSH, thyroid-stimulating hormone; FT4, free thyroxine; FT3, free triiodothyronine; and OR, odds ratio.

## Data Availability

All data generated or analyzed during this study are included in this article. The data will be available upon reasonable request (contact persons: osman.cure@erdogan.edu.tr and filiz.mercantepe@saglik.gov.tr).

## Supplementary Materials

The following supporting information can be downloaded at https://www.mdpi.com/article/10.3390/ijms26167862/s1.
