# Supplementary material for: Association of Low Free T3 with Disease Presence and Activity in Ankylosing Spondylitis
Source: Int J Mol Sci. 2025 Aug 14;26(16):7862. doi: 10.3390/ijms26167862 (PMC12386563; doi:10.3390/ijms26167862)
Supplement: Supplementary file 1 [file ijms-26-07862-s001.zip › ijms-3771155-supplementary.pdf]

Table S1. General data of the examined male population

	Control group<br>N=56	Ankylosing Spondylitis<br>N=74	*P*
Age, years	41.00 (33.50-50.50)	36.00 (30.00-44.00)	**0.022**
BMI, kg/m^2 #	29.89±4.72	26.03±4.21	**<0.001**
Waist circumference, cm	102.00 (96.00-108.50)	95.00 (89.00-106.00)	**0.004**
Diabetes mellitus (no/yes)	56/0	74/0	/
Coronary artery disease (no/yes)	56/0	73/1	0.383
Hypertension (no/yes)	53/3	73/1	0.190
Hyperlipidemia (no/yes)	56/0	73/1	0.383
Alcohol drinking status (no/yes)	53/3	67/7	0.385
Smoking status (no/yes)	32/24	37/37	0.419
Disease duration, months	/	7.50 (4.00-10.00)	/
BASDAI score	/	4.37±2.10	/
BASFI score	/	2.85 (1.00-4.90)	/

Data are presented as median (IQR) and compared with Mann-Whitney *U*-test.
Normally distributed data are presented as mean±standard deviation and compared with Student *t*-test.
Categorical variables are presented as absolute frequencies and compared by Chi-square test for contingency tables. Abbreviations: BMI, Body Mass Index; NSAID, Non-steroidal anti-inflammatory drugs; TNF-α - Tumor Necrosis Factor-alpha; BASDAI, Bath Ankylosing Spondylitis Disease Activity Index; BASFI, Bath Ankylosing Spondylitis Functional Index.

Table S2. Hematologic and biochemical markers of the examined male population

	Control group	Ankylosing Spondylitis	*P*
WBC count *1000	6.92 (5.68-8.73)	7.73 (6.75-9.15)	**0.013**
Neutrophil count *1000	3.73 (3.14-5.20)	4.71 (3.79-5.71)	**0.034**
Lymphocyte count *1000	2.36 (1.83-2.66)	2.47 (2.03-2.96)	0.140
Monocyte count *100	4.50 (3.65-5.90)	4.95 (4.30-6.30)	**0.043**
Platelet count *10000	26.00 (21.00-29.50)	27.50 (23.00-32.00)	**0.056**
Glucose, mg/dL	93.50 (89.00-100.00)	91.00 (85.00-99.00)	0.052
HbA1c, %	5.60 (5.40-5.95)	5.60 (5.20-5.80)	0.101
Urea (mg/dL)	29.00 (25.00-34.50)	27.50 (24.00-33.00)	0.327
Creatinine (mg/dL)	0.90 (0.82-1.00)	0.80 (0.70-0.90)	**0.003**
Uric acid (mg/dL)	5.80 (5.00-6.30)	5.70 (4.70-6.00)	0.225
Total protein (g/dL)	7.54 (7.40-7.80)	8.00 (7.20-8.00)	**0.038**
CRP (mg/L)	2.22 (1.00-3.60)	5.00 (2.00-10.00)	**0.001**
ESR (mm/hour)	4.50 (4.00-10.00)	9.00 (2.00-18.00)	0.149
Total cholesterol# (mg/dL)	224.64±42.84	200.03±37.98	**0.001**
HDL-C (mg/dL)	45.00 (39.00-52.30)	44.00 (38.00-51.00)	0.580
LDL-C# (mg/dL)	146.25±37.60	126.61±33.44	**0.002**
Triglycerides (mg/dL)	152.00 (89.50-199.00)	124.50 (86.00-178.00)	0.186
Vitamin B12 (pg/mL)	300.00 (264.00-382.00)	322.00 (259.00-406.00)	0.318
Folat (ng/mL)	8.71 (7.34-12.00)	8.00 (6.00-11.00)	0.104
Vitamin D (ng/mL)	8.84 (7.00-12.00)	11.50 (9.00-16.00)	**0.007**
Magnesium (mg/dL)	2.03 (1.91-2.14)	2.00 (1.90-2.10)	0.084
TSH (mIU/L)	2.10 (1.46-3.23)	1.52 (1.20-2.60)	**0.018**
FT4 (ng/dL)	1.20 (1.11-1.30)	1.21 (1.08-1.38)	0.799
FT3 (pg/mL)	3.60 (3.27-3.79)	3.43 (3.12-3.74)	*0.067*

Data are presented as median (interquartile range) and compared with Mann-Whitney *U*-test.
Normally distributed data are presented as arithmetic mean±standard deviation and compared with Student *t*-test. Abbreviations: WBC, White blood cell count; HbA1c, Hemoglobin A1c; CRP, C-reactive protein; ESR, Erythrocyte sedimentation

rate; HDL-C, High-density lipoprotein cholesterol; LDL-C, Low-density lipoprotein cholesterol; TSH, Thyroid-stimulating hormone; FT4, Free thyroxine; FT3, Free triiodothyronine.

Table S3. General data of the examined female population

	**Control group** N=61	**Ankylosing Spondylitis** N=46	*P*
Age, years	37.00 (29.00-43.00)	40.50 (34.00-51.00)	**0.017**
BMI, kg/m² #	27.03±5.11	27.48±6.46	0.683
Waist circumference, cm	86.00 (80.00-94.00)	88.00 (83.00-94.00)	0.231
Diabetes mellitus (no/yes)	61/0	45/1	0.246
Coronary artery disease (no/yes)	61/0	46/0	/
Hypertension (no/yes)	61/0	43/3	0.043
Hyperlipidemia (no/yes)	61/0	46/0	/
Alcohol drinking status (no/yes)	60/1	46/0	0.383
Smoking status (no/yes)	54/7	34/12	0.050
Disease duration, months	/	5.00 (3.00-8.00)	/
BASDAI score	/	4.35±2.10	/
BASFI score	/	3.70 (1.60-5.20)	/

Data are presented as median (IQR) and compared with Mann-Whitney *U*-test.
Normally distributed data are presented as mean±standard deviation and compared with Student *t*-test.
Categorical variables are presented as absolute frequencies and compared by Chi-square test for contingency tables. Abbreviations: BMI, Body Mass Index; BASDAI, Bath Ankylosing Spondylitis Disease Activity Index; BASFI, Bath Ankylosing Spondylitis Functional Index.

Table S4. Hematologic and biochemical markers of the examined female population

	**Control group**	**Ankylosing Spondylitis**	*P*
WBC count *1000	6.15 (5.18-87.61)	7.43 (6.01-8.58)	**0.002**
Neutrophil count *1000	3.54 (2.79-4.69)	4.37 (3.24-5.10)	0.063
Lymphocyte count *1000	1.93 (1.60-2.28)	2.11 (1.96-3.21)	**0.001**
Monocyte count *100	3.40 (2.80-3.90)	4.10 (3.50-5.10)	**<0.001**
Platelet count *10000	26.00 (22.00-30.00)	29.50 (28.00-34.00)	**<0.001**
Glucose, mg/dL	92.50 (86.00-96.00)	90.00 (84.00-97.00)	0.865
HbA1c, %	5.50 (5.30-5.70)	5.60 (5.10-6.00)	0.845
Urea (mg/dL)	23.00 (20.00-26.00)	24.00 (21.00-29.00)	0.219
Creatinine (mg/dL)	0.70 (0.65-0.78)	0.60 (0.50-0.70)	**<0.001**
Uric acid (mg/dL)	4.00 (3.40-4.70)	4.20 (3.60-5.00)	0.621
Total protein (g/dL)	7.60 (7.30-7.80)	7.60 (7.00-8.00)	0.415
CRP (mg/L)	1.56 (0.60-3.00)	4.00 (2.00-7.00)	**<0.001**
ESR (mm/hour)	8.00 (5.00-12.00)	15.50 (8.00-25.00)	**<0.001**
Total cholesterol# (mg/dL)	202.08±40.86	212.89±36.40	0.159
HDL-C (mg/dL)	58.00 (52.00-66.00)	56.00 (49.00-66.00)	0.227
LDL-C# (mg/dL)	121.77±34.54	132.91±30.85	0.087
Triglycerides (mg/dL)	86.00 (62.00-123.00)	100.50 (74.00-158.00)	0.102
Vitamin B12 (pg/mL)	304.00 (254.00-338.00)	340.00 (276.00-418.00)	**0.012**
Folat (ng/mL)	9.30 (7.32-11.13)	9.00 (8.00-11.00)	0.626
Vitamin D (ng/mL)	8.00 (5.00-16.28)	8.00 (5.70-13.00)	0.967
Magnesium (mg/dL)	2.00 (1.92-2.07)	1.90 (1.87-2.06)	0.080
TSH (mIU/L)	1.91 (1.47-2.89)	1.84 (1.34-3.36)	0.655
FT4 (ng/dL)	1.13 (1.00-1.18)	1.05 (1.00-1.22)	0.959
FT3 (pg/mL)	3.25 (3.08-3.47)	3.15 (2.65-3.26)	**0.009**

Data are presented as median (interquartile range) and compared with Mann-Whitney *U*-test.
Normally distributed data are presented as arithmetic mean±standard deviation and compared with Student *t*-test. Abbreviations: WBC, White blood cell count; HbA1c, Hemoglobin A1c; CRP, C-reactive protein; ESR, Erythrocyte sedimentation rate; HDL-C, High-density lipoprotein cholesterol; LDL-C, Low-density lipoprotein cholesterol; TSH, Thyroid-stimulating hormone; FT4, Free thyroxine; FT3, Free triiodothyronine.

Table S5. General data of male patients with Ankylosing Spondylitis according to BASDAI score

	BASDAI score < 4<br>N=33	BASDAI score ≥ 4<br>N=42	*P*
Age, years	36.00 (31.02-43.00)	36.00 (30.00-46.00)	0.749
BMI, kg/m² #	25.74±4.17	26.39±4.30	0.515
Waist circumference, cm	96.00 (91.00-101.00)	95.00 (89.00-110.00)	0.814
Diabetes mellitus (no/yes)	33/0	42/0	/
Coronary artery disease (no/yes)	33/0	41/1	0.372
Hypertension (no/yes)	33/0	41/1	0.372
Hyperlipidemia (no/yes)	33/0	41/1	0.372
Alcohol drinking status (no/yes)	29/4	38/4	0.718
Smoking status (no/yes)	16/17	21/21	0.896
Disease duration, months	10.00 (5.00-13.00)	6.00 (3.00-10.00)	0.050
NSAID/Anti-TNF-α agents	5/28	14/28	0.072
BASDAI score	2.50 (1.90-3.50)	5.50 (4.60-6.60)	**< 0.001**
BASFI score	1.00 (0.40-3.80)	4.15 (2.40-6.20)	**< 0.001**

Data are presented as median (interquartile range) and compared with Mann-Whitney *U*-test.
Normally distributed data are presented as mean±standard deviation and compared with Student *t*-test.
Categorical variables are presented as absolute frequencies and compared by Chi-square test for contingency tables. Abbreviations: BMI, Body Mass Index; NSAID, Non-steroidal anti-inflammatory drugs; TNF-*α* - Tumor Necrosis Factor-alpha; BASDAI, Bath Ankylosing Spondylitis Disease Activity Index; BASFI, Bath Ankylosing Spondylitis Functional Index.

Table S6. Hematologic and biochemical markers of male patients with Ankylosing Spondylitis according to BASDAI score

	BASDAI score < 4	BASDAI score ≥ 4	*P*
WBC count *1000	7.58 (6.60-9.05)	8.01 (6.95-9.55)	0.390
Neutrophil count *1000	4.17 (3.25-5.63)	4.86 (4.10-5.89)	0.195
Lymphocyte count *1000	2.56 (2.03-2.93)	2.41 (2.04-2.96)	0.940
Monocyte count *100	5.00 (4.60-6.70)	5.05 (4.20-6.10)	0.608
Platelet count *10000	27.00 (22.00-32.00)	28.00 (24.00-34.00)	0.560
Glucose (mg/dL)	92.00 (87.00-99.00)	91.00 (85.00-99.00)	0.642
HbA1c, %	5.65 (5.25-5.80)	5.35 (5.20-5.80)	0.394
Urea (mg/dL)	26.00 (23.00-29.00)	28.00 (24.00-33.00)	0.273
Creatinine (mg/dL)	0.80 (0.80-0.90)	0.82 (0.70-0.90)	0.935
Uric acid (mg/dL)	5.60 (4.60-6.00)	5.70 (4.80-6.00)	0.885
Total protein (g/dL)	8.00 (7.80-8.00)	7.60 (7.00-8.00)	0.075
CRP (mg/L)	3.70 (1.40-9.00)	6.00 (2.40-12.00)	0.182
ESR (mm/hour)	9.00 (2.00-18.00)	9.00 (3.00-17.00)	1.000
Total cholesterol# (mg/dL)	206.82±36.74	198.83±47.06	0.426
HDL-C (mg/dL)	47.00 (42.00-57.00)	40.50 (37.00-47.00)	**0.004**
LDL-C# (mg/dL)	129.76±34.37	128.24±34.37	0.867
Triglycerides (mg/dL)	130.00 (77.00-191.00)	124.50 (105.00-171.50)	0.848
Vitamin B12 (pg/mL)	361.00 (287.00-420.00)	296.00 (248.00-379.00)	0.027
Folat (ng/mL)	8.00 (6.90-13.00)	7.35 (6.00-10.00)	0.104
Vitamin D (ng/mL)	12.00 (9.00-16.00)	11.00 (9.00-17.00)	0.932

Magnesium (mg/dL)	2.00 (1.90-2.07)	2.00 (1.90-2.17)	0.516
TSH (mIU/L)	1.60 (1.10-2.47)	1.50 (1.20-2.60)	0.945
FT4 (ng/dL)	1.24 (1.10-1.33)	1.15 (1.08-1.39)	0.605
FT3 (pg/mL)	3.50 (3.36-3.87)	3.25 (3.02-3.51)	**0.010**

Data are presented as median (interquartile range) and compared with Mann-Whitney *U*-test.
Normally distributed data are presented as mean±standard deviation and compared with Student *t*-test. Abbreviations: WBC, White blood cell count; HbA1c, Hemoglobin A1c; CRP, C-reactive protein; HDL-C, High-density lipoprotein cholesterol; LDL-C, Low-density lipoprotein cholesterol; TSH, Thyroid-stimulating hormone; FT4, Free thyroxine; FT3, Free triiodothyronine.

Table S7. General data of female patients with Ankylosing Spondylitis according to BASDAI score

	BASDAI score < 4 N=11	BASDAI score ≥ 4 N=34	*P*
Age, years	41.00 (35.50-52.00)	39.00 (33.50-51.00)	0.643
BMI, kg/m^2 #	25.22±5.59	28.19±6.62	0.186
Waist circumference, cm	84.00 (83.00-90.00)	88.00 (83.00-100.50)	0.416
Diabetes mellitus (no/yes)	11/0	33/1	0.571
Coronary artery disease (no/yes)	11/0	34/0	/
Hypertension (no/yes)	9/2	33/1	0.078
Hyperlipidemia (no/yes)	11/0	34/0	/
Alcohol drinking status (no/yes)	11/0	34/0	/
Smoking status (no/yes)	7/4	26/8	0.403
Disease duration, months	5.00 (4.00-5.50)	5.00 (3.00-9.00)	0.659
NSAID/Anti-TNF-α agents	4/7	11/13	0.141
BASDAI score	3.30 (1.95-3.50)	5.80 (4.60-7.50)	**< 0.001**
BASFI score	1.60 (1.00-3.30)	3.95 (2.00-5.80)	**< 0.001**

Data are presented as median (interquartile range) and compared with Mann-Whitney *U*-test.
Normally distributed data are presented as mean±standard deviation and compared with Student *t*-test.
Categorical variables are presented as absolute frequencies and compared by Chi-square test for contingency tables. Abbreviations: BMI, Body Mass Index; NSAID, Non-steroidal anti-inflammatory drugs; TNF-α - Tumor Necrosis Factor-alpha; BASDAI, Bath Ankylosing Spondylitis Disease Activity Index; BASFI, Bath Ankylosing Spondylitis Functional Index.

Table S8. Hematologic and biochemical markers of female patients with Ankylosing Spondylitis according to BASDAI score

	BASDAI score < 4	BASDAI score ≥ 4	*P*
WBC count *1000	7.43 (6.47-7.80)	7.48 (5.97-9.02)	0.680
Neutrophil count *1000	4.90 (2.92-4.96)	4.33 (3.47-5.10)	0.589
Lymphocyte count *1000	2.09 (1.96-2.95)	2.17 (1.95-3.02)	1.000
Monocyte count *100	4.30 (4.05-4.55)	4.00 (3.40-5.30)	0.661
Platelet count *10000	32.00 (28.00-33.00)	29.00 (28.00-35.00)	0.765
Glucose (mg/dL)	84.00 (83.00-98.50)	91.00 (87.00-95.50)	0.285
HbA1c, %	5.40 (5.00-6.00)	5.50 (5.15-5.95)	0.964
Urea (mg/dL)	29.00 (24.00-31.50)	24.00 (20.50-28.00)	0.079
Creatinine (mg/dL)	0.60 (0.50-0.70)	0.60 (0.50-0.70)	0.840
Uric acid (mg/dL)	5.00 (4.15-5.00)	4.00 (3.40-4.55)	0.059
Total protein (g/dL)	7.60 (7.15-8.00)	7.50 (7.00-7.95)	0.785
CRP (mg/L)	5.00 (3.42-6.30)	3.90 (2.00-7.00)	0.806
ESR (mm/hour)	14.00 (8.00-19.00)	17.00 (10.50-26.00)	0.279
Total cholesterol# (mg/dL)	207.55±38.43	214.57±36.15	0.582
HDL-C (mg/dL)	58.00 (55.50-52.50)	54.00 (49.00-65.00)	0.241
LDL-C# (mg/dL)	123.09±27.01	136.00±31.62	0.230

Triglycerides (mg/dL)	83.00 (74.00-136.00)	101.00 (80.50-158.00)	0.562
Vitamin B12 (pg/mL)	336.00 (271.50-411.50)	345.00 (278.00-425.50)	0.817
Folat (ng/mL)	8.00 (6.50-9.50)	9.50 (8.00-11.00)	0.280
Vitamin D (ng/mL)	11.50 (6.00-20.00)	8.00 (5.55-11.50)	0.217
Magnesium (mg/dL)	1.90 (1.77-2.09)	1.90 (1.90-2.05)	0.647
TSH (mIU/L)	1.77 (1.58-2.17)	1.90 (1.28-3.58)	0.891
FT4 (ng/dL)	1.06 (0.95-1.31)	1.00 (1.01-1.20)	0.779
FT3 (pg/mL)	3.11 (2.36-3.38)	3.15 (2.70-3.22)	0.978

Data are presented as median (interquartile range) and compared with Mann-Whitney *U*-test.

Normally distributed data are presented as mean±standard deviation and compared with Student *t*-test. Abbreviations: WBC, White blood cell count; HbA1c, Hemoglobin A1c; CRP, C-reactive protein; HDL-C, High-density lipoprotein cholesterol; LDL-C, Low-density lipoprotein cholesterol; TSH, Thyroid-stimulating hormone; FT4, Free thyroxine; FT3, Free triiodothyronine.

Table S9. Comparison of thyroid functions according to disease duration.

	Disease duration ≤6 months (N=64)	Disease duration >6 months (N=55)	P
TSH (mIU/L)	1.60 (1.22-2.78)	1.64 (1.13-2.53)	0.484
FT4 (ng/dL)	1.12 (1.04-1.33)	1.17 (1.08-1.31)	0.935
FT3 (pg/mL)	3.20 (3.02-3.51)	3.36 (2.96-3.60)	0.786

Data are presented as median (interquartile range) and compared with Mann-Whitney *U*-test.

Normally distributed data are presented as mean±standard deviation and compared with Student *t*-test. Abbreviations: TSH, Thyroid-stimulating hormone; FT4, Free thyroxine; FT3, Free triiodothyronine.

Table S10. Comparison of thyroid functions according to treatment type.

	Patients receiving NSAIDs (N=45)	Patients receiving anti-TNF-α agents (N=75)	P
TSH (mIU/L)	1.62 (1.27-2.81)	1.62 (1.16-2.60)	0.566
FT4 (ng/dL)	1.15 (1.07-1.25)	1.07 (1.08-1.37)	0.098
FT3 (pg/mL)	3.20 (2.97-3.50)	3.30 (3.03-3.74)	0.187

Data are presented as median (interquartile range) and compared with Mann-Whitney *U*-test.

Normally distributed data are presented as mean±standard deviation and compared with Student *t*-test. Abbreviations: TSH, Thyroid-stimulating hormone; FT4, Free thyroxine; FT3, Free triiodothyronine.

Table S11. ROC analysis of FT3 discriminatory ability towards AS and high disease activity

Parameter	AUC	95% CI	P
FT3*	0.579	0.505-0.653	0.037
FT3**	0.659	0.554-0.764	0.004

Data are presented as Area under the curve (AUC) and 95% Confidence Interval (CI)

* Discriminatory ability towards AS

** Discriminatory ability towards high disease activity

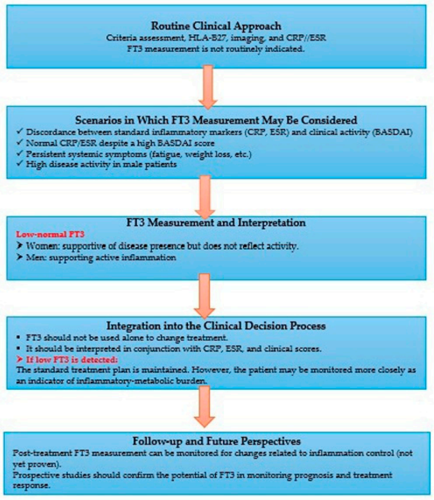

Figure S1. Proposed Exploratory Algorithm for the Potential Use of FT3 Measurement in Ankylosing Spondylitis Management.

This algorithm is exploratory and purely hypothetical, developed based on the findings of the present study. It is not intended for routine clinical use. Further prospective and interventional studies are required to validate its clinical applicability in ankylosing spondylitis.
